# Associations of formal childcare use with health and human capital development for adolescent mothers and their children in South Africa: A cross‐sectional study

**DOI:** 10.1111/cch.13138

**Published:** 2023-06-07

**Authors:** Lucie Cluver, Janina Jochim, Yolanda Mapukata, Camille Wittesaele, Yulia Shenderovich, Sandisiwe Mafuya, Kathryn Steventon Roberts, Bolade Banougnin, Lorraine Sherr, Elona Toska

**Affiliations:** ^1^ Department of Social Policy and Intervention University of Oxford Oxford UK; ^2^ Department of Psychiatry University of Cape Town Cape Town South Africa; ^3^ Teen Advisory Group University of Cape Town Cape Town South Africa; ^4^ Department of Infectious Disease Epidemiology London School of Hygiene and Tropical Medicine London UK; ^5^ Wolfson Centre for Young People's Mental Health University of Cardiff Cardiff UK; ^6^ Centre for the Development and Evaluation of Complex Interventions for Public Health Improvement (DECIPHer), School of Social Sciences Cardiff University Cardiff UK; ^7^ Institute for Global Health University College London London UK; ^8^ Centre for Social Science Research University of Cape Town Cape Town South Africa; ^9^ Department of Sociology University of Cape Town Cape Town South Africa

**Keywords:** adolescent mothers, adolescent pregnancy, human capital, school policies, South Africa

## Abstract

**Aim:**

This study aims to investigate associations of formal childcare with maternal and child outcomes in a large sample of adolescent mothers.

**Background:**

Forty percent of adolescent girls in Africa are mothers. Increasing evidence shows positive impacts of formal childcare use for adult women, but no known studies in the Global South examine associations for adolescent mothers and their children.

**Methods:**

We interviewed 1046 adolescent mothers and completed developmental assessments with their children (*n* = 1139) in South Africa's Eastern Cape between 2017 and 2019. Questionnaires measured childcare use, maternal and child outcomes and socio‐demographic background variables. Using cross‐sectional data, associations between formal childcare use and outcomes were estimated in multivariate multi‐level analyses that accounted for individual‐level and family‐level clustering.

**Results:**

Childcare use was associated with higher odds of being in education or employment (AOR: 4.01, 95% CIs: 2.59–6.21, *p* < .001), grade promotion (AOR: 2.08, 95% CIs: 1.42–3.05, *p* < .001) and positive future ideation (AOR: 1.58, 95% CIs: 1.01–2.49, *p* = .047) but no differences in mental health. Childcare use was also associated with better parenting on all measures: positive parenting (AOR: 1.66, 95% CIs: 1.16–2.38, *p* = .006), better parental limit‐setting (AOR: 2.00, 95% CIs: 1.37–2.93, *p* < .001) and better positive discipline (AOR: 1.77, 95% CIs: 1.21–2.59, *p* = .003). For the children, there were no differences in temperament or illness, but a significant interaction showed stronger associations between childcare use and better cognitive, language and motor scores with increasing child age (AOR: 5.04, 95% CIs: 1.59–15.96, *p* = .006).

**Conclusions:**

Adolescent mothers might benefit substantially from formal childcare, but causal links need to be explored further. Childcare use was also associated with improved parenting and better child development over time, suggesting positive pathways for children. At an average of $9 per month, childcare provisions for adolescent mothers may offer low‐cost opportunities to achieve high returns on health and human capital outcomes in Sub‐Saharan African contexts.

**Key Messages**

- Approximately one in three adolescent mothers reported using formal childcare services, paying an average of $9 a month.
- Multivariate multi‐level analyses identified associations of childcare use with improved education/employment outcomes, positive future outlook, parenting behaviours for young mothers and positive development for their children over time.
- International demands for affordable childcare should include considerations for adolescent parents, particularly in Sub‐Saharan Africa where rates of adolescent pregnancy remain high.
- Exploring the benefits of childcare for adolescent parents in different contexts, using different methodologies, is essential to cement calls for universal childcare as a cost‐effective and sustainable intervention.

## 1 INTRODUCTION

By the age of 20, 46% of adolescent girls in Sub‐Saharan Africa are already mothers (UNFPA, 2022), with emerging evidence suggesting that COVID's socio‐economic impacts have further increased adolescent pregnancy (Zulaika et al., 2022). Adolescent motherhood is frequently preceded by severe adversity, including poverty, orphanhood and child abuse (Thurman et al., 2006). Globally, adolescent motherhood is a marker of disadvantage, with higher levels of school dropout, unemployment, mental health challenges (Ardington et al., 2015; Huda et al., 2021) and lower future aspirations (Mchunu et al., 2012). In turn, adolescent parenthood predicts disadvantage in the next generation, with children of adolescent parents showing poorer health (Kurth et al., 2010), cognitive development and school readiness (Fall et al., 2015; Lipman et al., 2011). Pathways from maternal to child disadvantage have been examined—mostly in high‐income settings—identifying that adolescent parents have higher long‐term poverty, lower readiness for childrearing (Blum & Gates, 2015) and—often driven by poverty—lower parenting skills such as limit‐setting, cognitive stimulation and sensitivity with infants, compared with older parents (Barlow et al., 2011; Oxford & Spieker, 2006).

Despite the scale of the challenge, interventions in the region have focused on preventing teenage pregnancy, rather than on supporting adolescent parents (Mmari & Sabherwal, 2013). However, multiple systematic reviews of adolescent pregnancy prevention have shown no significant effects of any program in the Global South (DiCenso et al., 2002; Oringanje et al., 2016; Ramos, 2011). We lack an evidence base for services that can promote health and human capital development for adolescent mothers and their children.

For adult mothers, there is increasing international evidence that access to formal childcare for their children is associated with gains. Access to childcare is poorer in low‐income countries where about eight out of 10 children who need childcare do not have access to it (Devercelli & Beaton‐Day, 2020). Compared with high‐income countries, children from low‐income countries are five times less likely to be in childcare (Devercelli & Beaton‐Day, 2020). A recent study showed that, across 53 low‐ and‐middle‐income countries, 20% of children below age five were either left alone or left in the care of a sibling below the age of 10 for at least an hour per week (Samman et al., 2016). Data on the cost of formal childcare are limited, especially in the Global South (Devercelli & Beaton‐Day, 2020), but high cost remains a major concern for women in many Sub‐Saharan countries (Samman et al., 2016). Parents might also be discouraged by low‐quality childcare, which has been shown to affect child development negatively (Britto et al., 2017).

Centre‐based childcare has received limited attention in policy and research across low‐ and middle‐income countries (Bhan et al., 2020; Hughes et al., 2021). One recent study found that nearly half of working parents, and even one in five unemployed parents in a Nairobi slum, regularly used paid childcare (Clark et al., 2021). A systematic review of impacts of childcare provision for adult mothers in the Global South found increased maternal employment in all four identified studies (Evans et al., 2021). In Kenya, a randomized trial of the provision of subsidized childcare vouchers for adult women found an 8.5% increase in employment (Clark et al., 2019). In Brazil, Mexico and Guatemala, adult mothers with access to formal childcare had higher employment and self‐esteem (Angeles et al., 2014; Hallman et al., 2002; Sanfelice, 2019). In Mozambique, a randomized controlled trial of a preschool intervention increased labour supply of primary caregivers and improved some parenting skills (Martinez et al., 2012). This evidence does span a wide range of formal childcare services, from those with strong early childhood development curricula to low‐income small private childcare centres in the homes of local women. However, none of these studies included adolescent parents.

A systematic review of impacts of formal childcare use on child development (Brown et al., 2014) found only one study in the Global South, showing improved cognitive outcomes (Mwaura et al., 2008). Other evidence is mixed, with some studies showing no differences in child development (Angeles et al., 2014), whereas others show some improvements (Martinez et al., 2012; Mwaura et al., 2008). Several studies show no differences in child health (Nakahara et al., 2010). Again, none of the literature reported outcomes for the children of adolescent mothers, but there is some evidence from the Global North that formal childcare attendance may be associated with long‐term improved educational and economic outcomes (Domond et al., 2020).

Our scoping review,^1^ summarized above, identified no known research in the Global South that examines associations of access to formal childcare with outcomes for adolescent mothers, or the children of adolescent mothers. This study aims to start filling this gap through the following objective: to investigate associations of using formal childcare with maternal education, employment, future ideation, mental health and parenting and with child temperament, illness and cognitive, language and motor development in a large sample of adolescent mothers in South Africa's Eastern Cape Province.

## 2 METHODS

### 2.1 Patient and public involvement statement

Before data collection commenced, a Teen Advisory Group was set up, consisting of a group of adolescent mothers (all had their first child below the age of 19 but were up to 24 years old at the time of the baseline data collection). These young mothers provided feedback throughout the conceptual design of the study and gave input on the questionnaire items, design and interview practices. Two mothers were involved as co‐authors of this paper and commented on the analytical approach, study findings and interpretations.

### 2.2 Study design and sample size

This study used cross‐sectional data from the first wave of a prospective cohort study, collected between 2017 and 2019. A power analysis using G*Power 3.1.6 (Faul et al., 2009) used assumptions of 80% power, a Cohen's *f* .09 small effect size for maternal health outcomes, sub‐group analyses for HIV‐positive adolescent mothers and a conservatively estimated 10% attrition rate. This shows that a sample of at least 1000 adolescent mothers allowed for testing of the desired parameters (Jackson, 2003). The final sample consists of 1046 adolescent girls and young women (aged 10–24). All participants had their first pregnancy before the age of 20 (World Health Organization, 2020) but were aged up to 24 years at the time of data collection. We also collected data from the adolescent mothers' children (*n* = 1139) in addition to their completion of a developmental assessment.

### 2.3 Setting and sample

**FIGURE 1:**
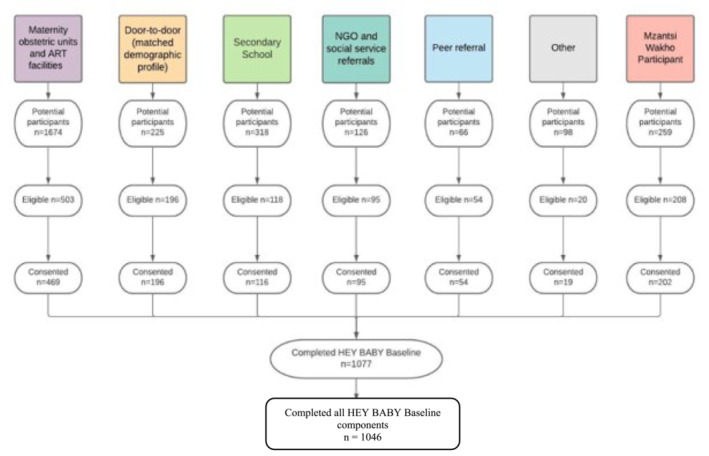
Recruitment flow diagram.

Adolescent girls and young women were recruited from urban and rural locations of two health districts in Eastern Cape South Africa. Based on formative work with an advisory group of adolescent mothers, six parallel recruitment channels were deployed to ensure the inclusion of hard‐to‐reach adolescents and to minimize recruitment bias. Recruitment took place through maternity obstetric units (*n* = 9) (UNFPA, 2022); all public district health facilities (*n* = 73) (Zulaika et al., 2022); a randomly selected subsample of secondary schools in the district (*n* = 43) (Thurman et al., 2006); referrals by social workers and NGOs (Ardington et al., 2015); door‐to‐door recruitment (Huda et al., 2021); and community referrals by adolescent mothers themselves (Mchunu et al., 2012), which were particularly valuable in areas with low service assess. Adolescent girls and young women were eligible for inclusion in the study if they were aged 10–24 at the time of the study, with at least one child who was conceived prior to the age of 20. Only participants residing in the Buffalo City Municipality or Amathole Health District were included in the study. Refusals and successful recruitment rates were recorded for each channel (see participant flowchart in Figure 1) showing that between 95% and 98% of all eligible mothers identified through each channel were successfully enrolled in the study.

### 2.4 Ethical considerations

This project involved working with a particularly vulnerable group. Adolescent pregnancy is associated with a range of adversities, including poverty, HIV risks and violence exposure. Our study site is based in the Eastern Cape, one of South Africa's poorest provinces where adolescent mothers have limited access to support sources and high rates of HIV pervade. Minimizing the risks for participants was an ethical imperative throughout the planning, data collection, storage, analysis and dissemination stages of all our research studies. Voluntary informed consent was sought from adolescents who were above the age of 18, and assent was provided by underage participants in addition to consent from their adult caregiver. All consent forms were available in English and Xhosa and were read aloud in cases of low literacy. Ethical approval was provided by the University of Oxford and the University of Cape Town (R48876/RE001; R48876/RE002; HREC REF: 226/2017) and the Eastern Cape Provincial Departments of Health, Basic Education, and Social Development.

### 2.5 Data collection procedures

Participants were interviewed in private spaces in and around their own home, or in a place of their choosing. Adolescents completed two questionnaires lasting about 60 min each, using audio mobile‐assisted self‐interviewing on electronic tablets, assisted by local interviewers trained to adjust the level of assistance by age and literacy abilities of participants. In addition, all children completed developmental assessments based on the Mullen Scales for Early Learning (Mullen, 1995), which took between 15 and 60 min, depending on the child's age and were scored by trained local research assistants. Please see Table 1 for a description of all items, scales and measures used in the study. Covariates for the analyses of maternal and parenting outcomes included *Age at pregnancy of the oldest child*, *age at the time of the interview*, *HIV status*, *co‐residency with a caregiver*, *rural/urban residency*, *orphanhood* (maternal or paternal), *living in informal housing* and *past‐week food insecurity*, *household size*, *childcare help received from the adolescent mother's own mother or caregiver*, *multiple children per adolescent mother*, and *child disability* as measured by the WHO ‘Ten Questions Screen’, which detects common disabilities, including physical, mental, speech, hearing, visual and epilepsy (Durkin et al., 1995). Analyses for child outcomes controlled for *child sex*, *age*, *adolescent mothers' highest achieved grade*, *child disability* and all maternal covariates.

**TABLE 1:** Scales and measures.

Construct	Items, measures and instruments
Formal childcare use	We asked participants if each of their children attend any childcare services. This included childcare centres (more formal registered centres), creches (usually local small businesses in homes) or reception classes (available in or around some primary schools). Because some adolescent mothers had multiple children, a binary variable was created that captured childcare use for all children in the family with ‘1’ (all children enrolled in formal childcare) or ‘0’ (one or more children *not* enrolled in formal childcare).
Adolescent mother outcomes	
School enrolment, tertiary education or employment	Items assessed if the participant was currently enrolled in education (secondary school, college or any further education/training) or engaged in any casual or permanent work. *Past year grade promotion* was captured with one item that assessed if mothers had passed a grade in the previous year (or had completed secondary education).
Positive future ideation	A series of items, co‐developed with participants, captured positive perceptions for having future good health, a satisfactory job, owning a house and stable financial situation. For each question, participants responded on a 4‐point scale from ‘very unlikely’ to ‘very likely’. The scale showed excellent internal consistency in the current sample (*α* = 0.95). A final binary variable was coded, capturing ‘yes’ (UNFPA, 2022) if participants responded ‘very likely’ to all four items.
Poor mental health	A binary item was coded based on participant scores above the cut‐off on any of three conditions: *Depression* within the previous 2 weeks, measured using the Child Depression Inventory short form (Kovacs, 1985). Scores of ≥3 (Allgaier et al., 2012; Greenland et al., 2016) were used to indicate symptomology for probable depression. The CDI‐S has previously been used with adolescent populations in South Africa (Cluver et al., 2007; Sharain, 2002; Shenderovich et al., 2021; Sherr et al., 2014; Woollett et al., 2017) and shows moderate psychometric properties (CDI‐S: *α* = 0.50 in the current sample). *Anxiety* over the previous month was measured using the Children's Manifest Anxiety Scale—Revised (Reynolds & Richmond, 1978), reduced to 14 items through factor analysis (Boyes et al., 2013). Scores of ≥10 were used to indicate anxiety symptomology (Gerard & Reynolds, 1999). The RCMAS has been validated within sub‐Saharan and shows good internal consistency among HIV‐affected children and adolescents (*α* = 0.86 in the current sample) (Boyes & Cluver, 2013). *Suicidality* and *self‐harm* in the past month were measured using the five‐item Mini International Neuropsychiatric Interview (Sheehan et al., 1997). Globally, the MINI‐KID has been extensively validated and demonstrates good internal consistency (*α* = 0.93 in the current sample) and good test–retest reliability (Sheehan et al., 1997; Sheehan et al., 2010). The final binary item classified participants as displaying any common poor mental health symptomology if they either reported (1) a score of ≥3 for probable depression or (2) scores of ≥10 indicating a positive screen for anxiety or (3) reported any suicidal symptoms.
Parenting outcomes	
Parenting	Parenting was assessed using an adapted version of the Parenting Young Children Scale (McEachern et al., 2012). After piloting the study questionnaire, we retained 15 items from the original measure that relate to three subscales: (UNFPA, 2022) positive parenting (e.g., ‘notice and praise your child's good behaviour’); (Zulaika et al., 2022) limit‐setting (e.g., ‘speak calmly with your child when you were upset with him/her’); and (Thurman et al., 2006) positive discipline (e.g., ‘plan ways to prevent problem behaviour’). For each question, the caregiver rated how often they were able to engage in the parenting behaviour on a 4‐point scale from ‘never’ to ‘almost daily’ (McEachern et al., 2012). Each subscale was coded as a binary, with ‘yes’ (UNFPA, 2022) if the participant responded ‘almost daily’ to all items. In a study of 6–12‐year‐olds in South Africa, the reliability of the scale was high: supporting positive behaviour (*α* = 0.78), setting limits (*α* = 0.79) and proactive parenting (*α* = 0.79) (Lester, 2015).
Child outcomes	
Child temperament	Child temperament was assessed using a shortened version (12 items) of the Emotionality, Activity and Shyness Temperament Questionnaire (EAS) that measures four behavioural dimensions: (1) shyness, (2) emotionality, (3) sociability and (4) activity (Buss & Plomin, 1984; Mathiesen & Tambs, 1999). Caregivers responded to three statements for each of the four domains, using a 5‐point scale ranging from 1 to 5. For each subscale, binary outcomes were coded as ‘yes’ (UNFPA, 2022) if the participant agreed to all items, indicating if children were active, social, not shy and able to control emotions. The validity and reliability of parent reports are consistently found to be good (Masi et al., 2003).
Child illness	Based on primary caregiver self‐report, a binary variable assessed if the child suffered from any serious illness in the previous year, such as pneumonia, pertussis, tuberculosis, meningitis or measles.
Child cognitive, language and motor development	Child development was assessed by trained data collectors using a scale based on the Mullen Scales of Early Learning (Mullen, 1995). These methods are described in more detail elsewhere (Steventon Roberts et al., 2022). *T*‐scores for four developmental domains—fine motor, visual reception, expressive language and receptive language—were combined and converted to age‐standardized *t*‐scores (range 45–155). These scores were used to create a composite score of generalized developmental functioning that was compared against a pooled estimate of means derived from nine sub‐Saharan African studies (see Knerr et al., 2013). Children scoring equal to or above the pooled average (*M* = 93.4; *SD* = 14.6) were coded ‘1’, whereas children with scores below the sample average were coded ‘0’.

Confidentiality was maintained throughout the study except where participants requested help or were at risk of significant harm. Twenty‐five referrals were made to health or counselling services with follow‐up support. There were no monetary incentives, but all participants received a certificate, refreshments and a participant pack containing items chosen by our adolescent advisory group (e.g., washcloth, soap and crayons).

### 2.6 Analyses

Adolescent mothers were eligible for inclusion in the analysis if they were pregnant within the ages of 10–19 (*n* = 16 excluded) and were the primary caregiver of a child (*n* = 71 excluded). An additional 31 participants were excluded because they showed missing values for the variables measuring childcare use (*n* = 13) and living in informal housing (*n* = 18). One hundred fifty‐two participants were excluded because they had children aged younger than 4 months, which is the standard maternity leave period in South Africa (Basic Conditions of Employment Act, 1997). Our final analyses of adolescent mothers were based on 776 adolescent mothers and 837 children. Analyses for the children's outcomes were restricted to children below 68 months, in line with the valid age range for the child development assessments using within this study, resulting in 25 children being excluded based on age.

Analyses were completed in STATA 15.1 and took place in six steps. The initial steps were completed to assess frequencies and distributions of all variables. In step 1, frequencies for all outcomes and covariates were examined, disaggregated by childcare use. In step 2, we evaluated univariable associations between childcare use and all outcomes for adolescent mothers and children, respectively. In step 3, we tested for associations between childcare use and all maternal and parenting outcomes. For this, we used a multivariate generalized estimating equations multi‐level analysis, which simultaneously estimated the effect of childcare use on all outcomes, controlling for a range of sociodemographic factors (the correlation matrix for all outcome variables is presented in Table 2). Using the xtgee command, the analyses account for within‐individual clustering of the outcomes by allowing for correlated residuals. We chose an unstructured correlation structure for the model as we did not have any prespecified assumptions about the within‐group correlation patterns. This approach provides an advantage compared to estimating each outcome in a separate regression model as it corrects potentially deflated standard errors because of outcomes that are clustered within individuals. In step 4, we tested for associations of childcare use with six child outcomes. Child outcomes and mother outcomes were estimated separately for two intertwined reasons. First, we did not seek to explore hypotheses on mother–child dyads (e.g., Rasbash et al., 2009). Second, we did not aim to account for correlations between all mother and child outcomes, although child analyses accounted for maternal socio‐demographic variables. In an initial exploration, we examined the within and between group variance, which showed that the clustering within the data requires a multi‐level approach to the analyses. The final model used a multivariate multi‐level analysis that simultaneously estimated all six child outcomes, controlling for key covariates pertaining to the mother and child (the correlation matrix for all outcome variables is presented in Table 3). Using the meglm command, the analyses account for two types of clustering allowing for correlated residuals. First, within‐individual clustering accounts for the clustering of all outcomes within the individual. Second, family‐level clustering addresses the household clustering of children of the same adolescent mother. Within these analyses, we included an interaction term between childcare use and child age as child development measures require adjustment by age to reflect developmental levels. In step 5, we estimated adjusted probabilities and marginal effects for experiencing each mother outcome, parenting outcome and child outcome, respectively, under two scenarios: not experiencing the hypothesized accelerator childcare use (UNFPA, 2022) and experiencing the hypothesized accelerator childcare use (Zulaika et al., 2022). In step 6, two sensitivity analyses were completed to supplement the robustness of the main findings.

**TABLE 2:** Bivariate correlations between outcome variables for adolescent mothers.

	School enrolment, tertiary education or employment	Grade promotion	Positive future ideation	Mental health symptomology	Positive parenting	Parental limit‐setting	Positive discipline
School enrolment, tertiary education and employment	‐						
Grade promotion	.62*	‐					
Positive future ideation	.42*	.32*	‐				
Mental health symptomology	−.19*	−.13	−.19*	‐			
Positive parenting	.03	.09	−.09	−.09	‐		
Parental limit‐setting	−.01	.10	−.14	−.15	.96*	‐	
Positive discipline	.01	.08	−.13	−.13	.92*	.98*	‐

* Significance level at < .05.

**TABLE 3:** Bivariate correlations between outcomes variables for children (shown only for the oldest children).

	Child temperament—emotionality	Child temperament—shyness	Child temperament—activity	Child temperament—sociability	Any severe illness, past year	Mullen child development scores
Child temperament—emotionality	‐					
Child temperament—shyness	.54*	‐				
Child temperament—activity	.67*	.57*	‐			
Child temperament—sociability	.18*	.40*	.40*	‐		
Any severe illness, past year	.03	.17	.19	.07	‐	
Mullen child development scores	.23*	.08	−.03	.8	.21	‐

* Significance level at < .05.

## 3 RESULTS

### 3.1 Descriptive data

Table 4 shows that the average age of participating adolescent mothers was 18.40 years (*SD* 1.72) at the time of the interview, and their mean age at birth of first child was 16.61 years (*SD* 1.55). Fifty‐nine adolescent mothers had given birth to two children (75% during adolescence), and three had given birth to three children at the time of the study. Nearly one third of participants lived in rural communities, and almost a quarter resided in informal housing. Just under 30% of adolescent mothers in the sample were living with HIV. About 40% of the adolescent mothers indicated being either maternally or paternally orphaned, but most adolescent mothers (91%) continued to live with a caregiver (either their biological parent or another adult) who provided some help with childcare (73.4%).

**TABLE 4:** Characteristics of adolescent mothers and children, overall and disaggregated by childcare use.

Adolescent mothers	*N* = 776% (*n*); *M* (*SD*)	*N* = 563% (*n*); *M* (*SD*)	*N* = 213% (*n*); *M* (*SD*)	*p*‐value
Age at first pregnancy, years	16.61 (1.55)	16.73 (1.55)	16.30 (1.49)	<.001
Current age, years	18.40 (1.72)	18.25 (1.73)	18.82 (1.61)	<.001
Living with HIV	29.12% (226)	28.60% (161)	30.52% (65)	.600
Adolescent mother lives with their caregiver	91.11% (707)	91.30% (514)	90.61% (193)	.780
Rural residency	29.77% (231)	31.62% (178)	24.88% (53)	.078
Orphanhood (maternal or paternal)	40.46% (314)	39.43% (222)	43.19% (92)	.370
Food insecurity (3 days/past week)	9.15% (71)	10.66% (60)	5.16% (11)	.017
Informal housing	22.42% (174)	23.27% (131)	20.19% (43)	.390
Household size	6.31 (2.68)	6.43 (2.68)	5.98 (2.66)	.037
Childcare help from caregiver	73.45% (570)	71.76% (404)	77.93% (166)	.084
Adolescent mothers with a disabled child	19.59% (152)	18.47% (104)	22.54% (48)	.220
School enrolment, tertiary education or employment	53.99% (419)	47.42% (267)	71.36% (152)	<.001
Grade promotion (past year)	57.60% (447)	53.99% (304)	67.14% (143)	<.001
Positive future ideation	79.12% (614)	77.44% (436)	83.57% (178)	.074
Positive parenting	36.98% (287)	32.68% (184)	48.36% (103)	<.001

First‐born children	*N* = 772% (*n*); *M* (*SD*)	*N* = 546% (*n*); *M* (*SD*)	*N* = 226% (*n*); *M* (*SD*)	*p*‐value
Sex				.058
Male	51.81% (400)	54.03% (295)	46.46% (105)	
Female	48.19% (372)	45.97% (251)	53.54% (121)	
Age, years	1.84 (1.24)	1.48 (1.06)	2.70 (1.23)	<.001
Disability	18.52% (143)	17.22% (94)	21.68% (49)	.150
Child temperament—emotionality	8.55% (66)	8.97% (49)	7.52% (17)	.570
Child temperament—shyness	4.66% (36)	4.95% (27)	3.98% (9)	.710
Child temperament—activity	9.59% (74)	7.88% (43)	13.72% (31)	.015
Child temperament—sociability	25.91% (200)	23.08% (126)	32.74% (74)	.007
Any severe illness, past year	1.35% (10)	0.95% (5)	2.29% (5)	.170
Child development scores, above sample average	48.32% (373)	50.18% (274)	43.81% (99)	.110

Second‐born children	*N* = 63% (*n*); *M* (*SD*)	*N* = 51% (*n*); *M* (*SD*)	*N* = 12% (*n*); *M* (*SD*)	
Sex				1.00
Male	36.51% (23)	37.25% (19)	33.33% (4)	
Female	63.49% (40)	62.75% (32)	66.67% (8)	
Age, years	1.58 (1.02)	1.35 (0.89)	2.55 (1.02)	<.001
Disability	19.05% (12)	21.57% (11)	8.33% (1)	.430
Child temperament—emotional	9.52% (6)	11.76% (6)	0.00% (0)	.590
Child temperament—shyness	4.76% (3)	5.88% (3)	0.00% (0)	1.00
Child temperament—activity	9.52% (6)	9.80% (5)	8.33% (1)	1.00
Child temperament—sociability	28.57% (18)	23.53% (12)	50.00% (6)	.085
Any severe illness, past year	0.00% (0)	0.00% (0)	0.00% (0)	‐
Child development scores, above sample average	42.86% (27)	45.10% (23)	33.33% (4)	.530

Third‐born children	*N* = 2% (*n*); *M* (*SD*)	*N* = 0% (*n*); *M* (*SD*)	*N* = 2% (*n*); *M* (*SD*)	
Sex		‐		‐
Male	50.00% (1)	‐	50.00% (1)	
Female	50.00% (1)	‐	50.00% (1)	
Age, years	2.4 (1.06)	‐	2.4 (1.06)	‐
Disability	0% (0)	‐	0% (0)	‐
Child temperament—emotional	0% (0)	‐	0% (0)	‐
Child temperament—shyness	0% (0)	‐	0% (0)	‐
Child temperament—activity	0% (0)	‐	0% (0)	‐
Child temperament—sociability	0% (0)	‐	0% (0)	‐
Any severe illness, past year	0% (0)	‐	0% (0)	‐
Child development scores, above sample average	50% (1)	‐	50% (1)	‐

The profiles of all participating children in the study are summarized in Table 4. Proportions of boys and girls were similar. There were no gender differences in formal childcare use for boys and girls. First‐born children had a mean age of 1.84 years (*SD* 1.24).

### 3.2 Multivariable associations between childcare use and outcomes for adolescent mothers

**FIGURE 2:**
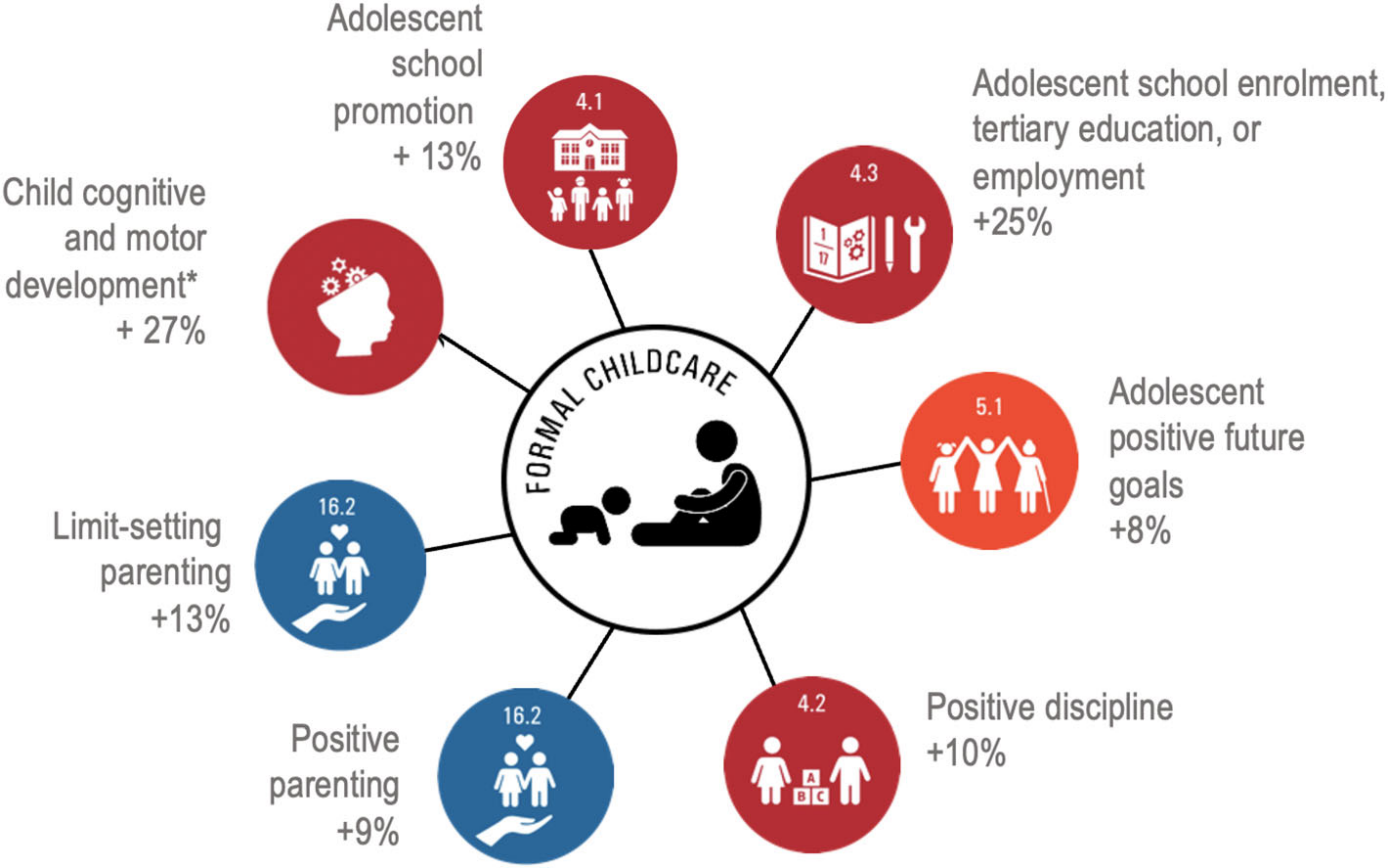
Model effects of formal childcare use on multiple outcomes.

Formal childcare use was associated with significantly higher odds of mothers being enrolled in secondary school, tertiary education or engaged in employment (AOR: 4.01, 95% CIs: 2.59–6.21, *p* < .001), higher odds of grade promotion (AOR: 2.08, 95% CIs: 1.42–3.05, *p* < .001) and higher odds for positive future ideation (AOR: 1.58, 95% CIs: 1.01–2.49, *p* = .047) (Figure 2 and Table 5). Adjusted probabilities are shown in Table 6. Without formal childcare use, the adjusted probability for school enrolment, tertiary education or employment was 47%, rising to 71% with formal childcare use. Without formal childcare use, the adjusted probability for grade promotion was 53%, rising to 68% with formal childcare use. Without formal childcare use, the adjusted probability for positive future goals was 77%, rising to 84% with formal childcare use.

**TABLE 5:** Multivariable associations between formal childcare use and outcomes for adolescent mothers and children.

	Adjusted odds ratio	Std. err.	*z*	*P* > |*z*|	[95% Conf. Interval]	
**Adolescent mother outcomes**						
School enrolment, tertiary education or employment	4.01	.89	6.24	<.001	2.59	6.21
Grade promotion	2.08	.40	3.76	<.001	1.42	3.05
Positive future ideation	1.58	.37	1.99	.047	1.01	2.49
Mental health symptomology	1.44	.37	1.40	.160	.87	2.38
Positive parenting	1.66	.30	2.77	.006	1.16	2.38
Parental limit‐setting	2.00	.39	3.58	<.001	1.37	2.93
Positive discipline	1.77	.34	2.95	.003	1.21	2.59
**Child outcomes**						
Child temperament—emotionality	.44	.39	−0.92	.358	.08	2.50
Child temperament—shyness	.30	.33	−1.08	.278	.03	2.62
Child temperament—activity	1.74	1.33	0.72	.469	.39	7.78
Child temperament—sociability	2.20	1.41	1.23	.217	.63	7.75
Any severe illness, past year	.45	.78	−0.51	.612	.02	9.35
Child development scores, above pooled average from the region	.04	.06	−2.27	.023	.00	.65

**TABLE 6:** Adjusted probabilities (marginal effects) and risk differences for adolescent mothers' outcomes, disaggregated by childcare use and non‐use.

	School enrolment, tertiary education or employment		Grade promotion		Positive future ideation		Any mental health symptomology		Positive parenting		Parental limit‐setting		Positive discipline	
	Adjusted probability (95% CI)	Probability difference (95% CI)	Adjusted probability (95% CI)	Probability difference (95% CI)	Adjusted probability (95% CI)	Probability difference (95% CI)	Adjusted probability (95% CI)	Probability difference (95% CI)	Adjusted probability (95% CI)	Probability difference (95% CI)	Adjusted probability (95% CI)	Probability difference (95% CI)	Adjusted probability (95% CI)	Probability difference (95% CI)
Childcare use														
No	0.47 [0.43–0.51]	‐	0.53 [0.49–0.57]	‐	0.77 [0.74–0.81]	‐	0.12 [0.09–0.15]	‐	0.34 [0.29–0.38]	‐	0.24 [0.20–0.27]	‐	0.25 [0.21–0.28]	‐
Yes	0.71 [0.66–0.77]	**0.25** *** **[0.18–0.33]**	0.68 [0.63–0.75]	**0.13** *** **[0.06–0.20]**	0.84 [0.79–0.89]	0.08* [−0.00–0.17]	0.16 [0.11–0.21]	0.06 [−0.03–0.16]	0.45 [0.38–0.52]	**0.09** ** **[0.03–0.16]**	0.38 [0.31–0.44]	**0.13** *** **[0.06–0.19]**	0.36 [0.29–0.43]	**0.10** ** **[0.03–0.17]**

*** Significance level at < .001.

** Significance level at < .01.

* Significance level at < .05.

### 3.3 Multivariable associations between childcare use and parenting for adolescent mothers

Formal childcare use was associated with better parenting behaviours on all measures: higher levels of positive parenting (AOR: 1.66, 95% CIs: 1.16–2.38, *p* = .006), better parental limit‐setting (AOR: 2.00, 95% CIs: 1.37–2.93, *p* < .001) and better positive discipline (AOR: 1.77, 95% CIs: 1.21–2.59, *p* = .003) (Figure 2 and Table 5). Without formal childcare use, the adjusted probability for positive parenting was 34%, rising to 45% with formal childcare use. The adjusted probability for parental limit‐setting rose from 24% to 38%, and the adjusted probability for positive discipline rose from 25% to 36% (see Table 6).

### 3.4 Multivariable associations between formal childcare use and outcomes for children of adolescent mothers

Formal childcare use was not associated with any child temperament measures, nor any severe illness, but it was associated with scores of the development of the children (AOR: 0.04, 95% CIs: 0.00–0.65, *p* = .023) (Figure 2 and Table 5). The interaction term between formal childcare use and child age on these scores showed that children attending childcare had better developmental scores as they grew older, compared with those who did not attend childcare (AOR: 5.04, 95% CIs: 1.59–15.96, *p* = .006). Based on these results, we calculated adjusted probabilities for the child developmental outcomes for different ages. Table 7 shows that, without formal childcare use, the adjusted probability for higher development scores was 49%, falling to 37% with formal childcare use for children aged one. There were no significant differences between the adjusted probability for child development with childcare use (36%) versus without formal childcare use (35%) at age 2. At age 3, without childcare use, the adjusted probability for higher developmental scores was 23%, rising to 35% with childcare use. At age 4, without childcare use, the adjusted probability for higher developmental scores was 14%, rising to 33% with childcare use. At age 5, without formal childcare use, the adjusted probability for higher development scores was 7%, rising to 32% with childcare use. At age 6, without childcare use, the adjusted probability for higher developmental scores was 4%, rising to 31% with childcare use.^2^

**TABLE 7:** Adjusted probabilities (marginal effects) and risk differences for children's cognitive, language and motor outcomes, disaggregated by childcare use and non‐use for different child ages.

	1 year old		2 years old		3 years old		4 years old		5 years old		6 years old	
	Adjusted probability (95% CI)	Probability difference (95% CI)	Adjusted probability (95% CI)	Probability difference (95% CI)	Adjusted probability (95% CI)	Probability difference (95% CI)	Adjusted probability (95% CI)	Probability difference (95% CI)	Adjusted probability (95% CI)	Probability difference (95% CI)	Adjusted probability (95% CI)	Probability difference (95% CI)
Childcare use												
No	0.49 [0.42–0.55]	‐	0.35 [0.30–0.39]	‐	0.23 [0.15–0.12]	‐	0.14 [0.05–0.23]	‐	0.07 [−0.00–0.15]	‐	0.04 [−0.02–0.09]	‐
Yes	0.37 [0.25–0.48]	**0.12** * [0.00–0.24]	0.36 [0.28–0.43]	−0.1 [0.09–0.07]	0.35 [0.26–0.43]	**−0.12** ** [−0.02–−0.20]	0.33 [0.20–0.47]	**−0.19** *** [−0.07–0.31]	0.32 [0.13–0.51]	**−0.25** *** [−0.08–0.41]	0.31 [0.06–0.55]	**−0.27** ** [−0.05–0.49]

*** Significance level at < .001.

** Significance level at < .01.

* Significance level at < .05.

## 4 DISCUSSION

In 2020, a report by the World Bank found that access to quality childcare has the potential to unlock pathways out of poverty, build human capital amongst women and their children and increase inclusive growth at a national level. It identified deeply uneven access in childcare provision and recommended that a crucial component of the COVID recovery will be smart public finance investments to support the childcare industry (Devercelli & Beaton‐Day, 2020). However, the report identified almost no evidence focused on adolescent parents.

This study provides some of the first evidence from the Global South of associations between access to formal childcare and maternal, parenting and child outcomes for adolescent mothers. It finds substantial positive associations for adolescent mothers of having children in formal childcare, particularly on maternal access to education and employment, grade promotion and positive future ideation. This is crucial for adolescent health and human capital outcomes: Improving educational access and positive future ideation are predictive of improved lifetime employment, earnings, better birth spacing, reduced intimate partner violence, HIV incidence and better child health (Abramsky et al., 2011; Balaj et al., 2021; Gadoth & Heymann, 2020; Schultz, 2002; Vandemoortele & Delamonica, 2000).

This study also finds positive associations of access to childcare across all measured parenting outcomes: positive parenting, parental limit‐setting and positive discipline. Improved early parenting is associated with better outcomes for children, with higher school readiness, executive function, lifetime earnings (Shafiq et al., 2018) and lower behaviour problems (Black et al., 2017; Richter et al., 2017). In particular, parental warmth and positive discipline/limit setting are identified with reduced rates of family violence against children, and protective parent–child relationships (Knerr et al., 2013). Access to stable childcare could affect parenting via reduced parenting stress (Abidin, 1992; Crnic et al., 2005; Deater‐Deckard, 1998), even though there is mixed evidence on this relationship (Craig & Churchill, 2018). Young mothers from our Teen Advisory Group confirmed the importance of childcare for their capabilities of education, employment and future aspirations.

The study did not find differences on maternal mental health, or on child temperament. This reflects mixed evidence on adult mothers and their children but may also highlight important contextual challenges. Adolescent motherhood (particularly out of marriage) remains highly stigmatized globally (Hall et al., 2018), and studies in the region report high levels of emotional distress experienced by adolescent mothers (Mutahi et al., 2022). Young mothers in our Teen Advisory Group echoed the concern that young mothers likely require additional support to cope with early parenthood, frequently co‐occurring with rejection from families and the child's father, and with postpartum mental health challenges. They also identified that formal childcare led to both improved child behaviour (through teachers) and potential behaviour challenges (through contact with other children)—reflecting wider literature on school and child behaviour in low‐income areas.

However, our findings show that children attending childcare have better cognitive, language and motor outcomes as they age, compared with children not attending childcare. This is of crucial importance for a group highly vulnerable to poor educational outcomes. Systematic reviews of child outcomes identify that quality of childcare (e.g., stimulation of children) may determine whether there are beneficial impacts on child health and development (National Institute of Child Health and Human Development Early Child Care Research Network & Duncan, 2003). Even in the very deprived setting of South Africa's Eastern Cape, where childcare services are most often in local homes and lacking qualified staff, we see positive outcomes, but this also suggests the added value and potential for building capacity and resources in early childhood development within childcare settings (Wolf et al., 2019). In our study, adolescent mothers reported the average cost for formal childcare to be 154 South African Rand per month, which converts to about $9, rendering it a low‐cost option to improve outcomes for adolescents and children. Previous research showed that, across 73 developing countries, increasing preschool enrolment to 50% in a single year could increase a countries' productivity by $33 billion across those children's lifetimes (Engle et al., 2011). Evidence‐based multi‐sectoral packages, which could include access to childcare, for pregnant adolescents and adolescent mothers are a crucial step in supporting their school progression and HIV risk reduction, strengthening positive future outlook and supporting the healthy development for the next generation (Cluver et al., 2022; Jochim et al., Forthcoming). There are important future areas for research in understanding associations of different kinds of childcare settings with child and adolescent mother outcomes and differential impacts by age of children. Following others' recommendation on childcare in Sub‐Saharan Africa (Bakibinga & Matanda, 2022), future research should also further assess the current obstacles of adolescent mothers to access childcare and interrogate how to better connect young mothers to existing childcare services. We need more rigorous research on the effective ingredients for support packages serving adolescent mothers and comprehensive evidence syntheses with multiple stakeholders to start developing pilot packages that can be tested in communities (Kelly et al., 2023). Additional costing analyses are necessary to encourage the allocation of financial resources from the government towards quality early care that lead to a move away from informal centres and overburdening families with childcare cost. Our correlational findings also warrant further testing of causal relationships between formal childcare use and adolescent and child outcomes through randomized controlled trials and investigations into the pathways towards important development outcomes. These explorations should include how to effectively measure quality service provisions and identify specific objectives for parents, households, communities and child‐level outcomes.

This study has several limitations. First, we used cross‐sectional data that limit the capacity to determine causality and, for some of the identified relationships, reverse causality is possible. For example, mothers who are well supported to return to school or remain employed after the pregnancy might be more likely to use childcare. However, analyses controlled for factors likely to influence childcare access, maternal and child outcomes, such as poverty, informal housing, maternal age, HIV status, household composition, number of children and child disability. Second, there is always risk of social desirability bias, which may have affected self‐reported parenting by adolescent mothers (although this was unlikely to differ by childcare access), and some of the items on parenting may have been less applicable to very young children. We attempted to reduce these limitations through pre‐piloting our questionnaire items with the Teen Advisory Group and highly empathetic, very well trained, local interviewers, many of whom had been young parents themselves. We conducted sensitivity analyses restricted to children over 12 months old, finding similar associations despite reduced sample size. Lastly, the generalizability to other low‐ and middle‐income country settings remains unknown, and further testing is clearly required, although the socio‐economic conditions and limited service access in the study area may be representative of conditions across Southern Africa (see Tables 8 and 9).

**TABLE 8:** Sensitivity analyses: Multivariable associations between childcare use and outcomes for mothers with children who are above the age of 1 year (*n* = 507).

	Adjusted odds ratio	Std. err.	*z*	*P* > |*z*|	[95% Conf. Interval]	
School enrolment and employment	3.63	.88	5.31	<.001	2.25	5.83
Grade promotion	2.25	.48	3.81	<.001	1.48	3.43
Positive future ideation	1.71	.43	2.14	.033	1.05	2.79
Mental health symptomology	1.50	.48	1.37	.170	.84	2.69
Positive parenting	1.46	.29	1.90	.057	.99	2.17
Parental limit‐setting	1.67	.35	2.41	.016	1.10	2.52
Positive discipline	1.46	.31	1.80	.072	.96	2.21

*Note*: The table below shows consistency with our main results, indicating that formal childcare use was associated with significantly higher odds of maternal school enrolment, tertiary education or employment (AOR: 3.63, 95% CIs: 2.25–5.83, *p* < .001), higher odds for grade promotion (AOR: 2.25, 95% CIs: 1.48–3.43, *p* < .001), and higher odds for positive future goals (AOR: 1.71, 95% CIs: 1.05–2.79, *p* = .033). Formal childcare use was also associated with higher odds for better parental limit‐setting (AOR: 1.67, 95% CIs: 1.10–2.52, *p* = .02), and similar trends (but not reaching significance) for positive parenting (AOR: 1.46, 95% Cis: 0.99–2.17, *p* = .057) and increased positive discipline (AOR: 1.46, 95% CIs: 0.96–2.21, *p* = .07).

**TABLE 9:** Sensitivity analyses: Multivariable associations between childcare use and outcomes for children who are above the age of 1 year (*n* = 588).

	Adjusted odds ratio	Std. err.	*z*	*P* > |*z*|	[95% Conf. Interval]	
Child temperament—emotionality	.72	.30	−0.78	.436	.32	1.63
Child temperament−shyness	.50	.28	−1.22	.221	.17	1.50
Child temperament−activity	1.17	.41	0.46	.648	.59	2.34
Child temperament−sociability	1.30	.34	1.03	.305	.78	2.16
Any severe illness, past year	.76	.57	−0.25	.725	.18	3.33
Mullen child development scores, above pooled average from the region	1.73	1.04	0.92	.345	.53	5.62

*Note*: For the children, the table below shows results for all child outcomes for participants whose children were above 1 year. The results reflect previous analyses, showing no significant differences for any child temperament scales or child illness. These supplementary analyses did not show a significant interaction between formal childcare use and child age for child development once we excluded children below 1 year.

The study also has key strengths. To our knowledge, it is the first large‐scale study of adolescent mothers and their children in the Global South and included independent cognitive, language and motor development testing of their children. The study took place in a real‐world, highly deprived setting, allowing an understanding of associations of childcare access even in a context where this was likely to be lower quality than that provided in randomized trials. This study used analysis techniques that allowed simultaneous modelling of correlated outcomes within mothers, such as educational success and positive future ideation, and children, such as illness and child development.

Quality formal childcare provision is emerging as a global need, with the International Labor Organization estimating that 800 million children under 6 years old need access to care (Addati et al., 2018). UN Women highlight the potential ‘triple dividend’ of increasing female labour force participation, improving child outcomes and creating jobs in the care sector (UN WOMEN, 2015). Particularly vulnerable adolescents and young women, such as those working in the informal economy and adolescent mothers, might share a particular need for childcare support (Horwood et al., 2019). The COVID pandemic—where billions of families struggled to manage without childcare access—has highlighted its importance in the world economy (Gromada et al., 2020). Our findings suggest that adolescent mothers and their children are an essential group to consider in the increasing demand for access to childcare. Improving access to childcare services represents an important intervention that needs to be further explored with different methodologies, considering causal impact (Richardson et al., 2018). In regions like Sub‐Saharan Africa, they are a major population group, experiencing severe intergenerational disadvantage and future risk. Providing access to reliable, subsidized quality childcare may be an essential ingredient in improving health and human capital for adolescent mothers and their children.

## CONFLICT OF INTEREST STATEMENT

The authors declare no conflict of interest.

## DATA SHARING AND DATA AVAILABILITY STATEMENT

Prospective users, policymakers/government agencies/researchers (internal/external) will be required to contact the study team to discuss and plan the use of data. Research data will be available on request subject to participant consent and having completed all necessary documentation. All data requests should be sent to the Principal Investigator.

## ETHICS APPROVAL STATEMENT

Ethical approval was provided by the University of Oxford and the University of Cape Town (R48876/RE001; R48876/RE002; HREC REF: 226/2017) and the Eastern Cape Provincial Departments of Health, Basic Education, and Social Development.

## PARENT CONSENT STATEMENT

Voluntary informed consent was sought from adolescents who were above the age of 18 and assent was provided by underage participants in addition to consent from their adult caregiver.

## Endnotes

^1^
A scoping review was led by two co‐authors. Searches were conducted on Google Scholar and PubMed during January–March 2022. The search strategy aimed to identify any research or program report examining the effect of a range of formal childcare services (e.g., universal childcare, day care, creche, playschool and center‐based day care) on any adolescent mother outcomes including mental health, school enrolment, future ideation and aspirations, health, household income and parental employment. Publications were screened based on relevance of the title and reference lists of relevant publications were additionally screened.

^2^
Parenting Young Children Scale includes items that might not apply to very young children, and some children may enter childcare later (i.e., after 12 months). Therefore, we conducted sensitivity analyses for a subsample of participants whose children are above 1 year old. The results indicate consistent results.

## References

[cch13138-bib-0001] Abidin, R. R. (1992). The determinants of parenting behavior. Journal of Clinical Child Psychology, 21, 407–412. 10.1207/s15374424jccp2104_12

[cch13138-bib-0002] Abramsky, T. , Watts, C. H. , Garcia‐Moreno, C. , Devries, K. , Kiss, L. , Ellsberg, M. , Jansen, H. A. F. M. , & Heise, L. (2011). What factors are associated with recent intimate partner violence? Findings from the WHO multi‐country study on women's health and domestic violence. BMC Public Health, 11(1), 109. 10.1186/1471-2458-11-109 21324186 PMC3049145

[cch13138-bib-0003] Addati, L. , Cattaneo, U. , Esquivel, V. , & Valarino, I. (2018 [cited 2022 Jun 14]). International labour office. Care work and care jobs for the future of decent work [Internet]. International Labour Office. Available from: https://search.ebscohost.com/login.aspx?direct=true&scope=site&db=nlebk&db=nlabk&AN=3109795

[cch13138-bib-0004] Allgaier, A. K. , Frühe, B. , Pietsch, K. , Saravo, B. , Baethmann, M. , & Schulte‐Körne, G. (2012). Is the children's depression inventory short version a valid screening tool in pediatric care? A comparison to its full‐length version. Journal of Psychosomatic Research, 73(5), 369–374. 10.1016/j.jpsychores.2012.08.016 23062811

[cch13138-bib-0005] Angeles, G. , Gadsen, P. , Galiani, S. , Gertler, P. , Herrera, A. , Kariger, P. , & Seira, E. (2014 [cited 2022 Jun 15]). The impact of daycare on maternal labour supply and child development in Mexico [Internet]. 3ie Impact Evaluation Report 6. New Delhi: International Initiative for Impact (3ie). Available from: https://www.3ieimpact.org/evidence-hub/publications/impact-evaluations/impact-daycare-maternal-labour-supply-and-child

[cch13138-bib-0006] Ardington, C. , Menendez, A. , & Mutevedzi, T. (2015). Early childbearing, human capital attainment and mortality risk: Evidence from a longitudinal demographic surveillance area in rural‐KwaZulu‐Natal, South Africa. Economic Development and Cultural Change, 63(2), 281–317. 10.1086/678983 26028690 PMC4443483

[cch13138-bib-0007] Bakibinga, P. , & Matanda, D. J. (2022 [cited 2023 Mar 21]). The salutogenic approach to childcare in Sub‐Saharan Africa: A focus on children who thrive in the face of adversity. In M. B. Mittelmark , G. F. Bauer , L. Vaandrager , J. M. Pelikan , S. Sagy , M. Eriksson , et al. (Eds.), The handbook of salutogenesis [Internet] (pp. 495–501). Springer International Publishing. Available from: 10.1007/978-3-030-79515-3_46 36121998

[cch13138-bib-0008] Balaj, M. , York, H. W. , Sripada, K. , Besnier, E. , Vonen, H. D. , Aravkin, A. , Friedman, J. , Griswold, M. , Jensen, M. R. , Mohammad, T. , Mullany, E. C. , Solhaug, S. , Sorensen, R. , Stonkute, D. , Tallaksen, A. , Whisnant, J. , Zheng, P. , Gakidou, E. , & Eikemo, T. A. (2021). Parental education and inequalities in child mortality: A global systematic review and meta‐analysis. The Lancet, 398(10300), 608–620. 10.1016/S0140-6736(21)00534-1 PMC8363948 34119000

[cch13138-bib-0009] Barlow, J. , Smailagic, N. , Bennett, C. , Huband, N. , Jones, H. , & Coren, E. (2011). Individual and group based parenting programmes for improving psychosocial outcomes for teenage parents and their children. Cochrane Database of Systematic Reviews, 16(3), CD002964. 10.1002/14651858.CD002964.pub2 PMC4164375 21412881

[cch13138-bib-0010] Basic Conditions of Employment Act , Government Gazette 1 [Internet]. 1997. Available from: https://www.gov.za/sites/default/files/gcis_document/201409/a75-97.pdf

[cch13138-bib-0011] Bhan, G. , Surie, A. , Horwood, C. , Dobson, R. , Alfers, L. , Portela, A. , & Rollins, N. (2020). Informal work and maternal and child health: A blind spot in public health and research. Bulletin of the World Health Organization, 98(3), 219–221. 10.2471/BLT.19.231258 32132757 PMC7047022

[cch13138-bib-0012] Black, M. M. , Walker, S. P. , Fernald, L. C. H. , Andersen, C. T. , DiGirolamo, A. M. , Lu, C. , McCoy, D. , Fink, G. , Shawar, Y. R. , Shiffman, J. , Devercelli, A. E. , Wodon, Q. T. , Vargas‐Barón, E. , Grantham‐McGregor, S. , & Lancet Early Childhood Development Series Steering Committee . (2017). Early childhood development coming of age: Science through the life course. The Lancet, 389(10064), 77–90. 10.1016/S0140-6736(16)31389-7 PMC5884058 27717614

[cch13138-bib-0013] Blum, R. W. , & Gates, W. H. (2015 [cited 2022 Jun 14]). Girlhood, not motherhood. Preventing adolescent pregnancy [Internet]. United Nations Population Fund (UNFPA). Available from: https://www.unfpa.org/sites/default/files/pub-pdf/Girlhood_not_motherhood_final_web.pdf

[cch13138-bib-0014] Boyes, M. E. , & Cluver, L. D. (2013). Performance of the revised children's manifest anxiety scale in a sample of children and adolescents from poor urban communities in Cape Town. European Journal of Psychological Assessment, 29(2), 113–120. 10.1027/1015-5759/a000134

[cch13138-bib-0015] Boyes, M. E. , Mason, S. J. , & Cluver, L. D. (2013). Validation of a brief stigma‐by‐association scale for use with HIV/AIDS‐affected youth in South Africa. AIDS Care, 25(2), 215–222. 10.1080/09540121.2012.699668 22774842

[cch13138-bib-0016] Britto, P. R. , Lye, S. J. , Proulx, K. , Yousafzai, A. K. , Matthews, S. G. , Vaivada, T. , Perez‐Escamilla, R. , Rao, N. , Ip, P. , Fernald, L. C. H. , MacMillan, H. , Hanson, M. , Wachs, T. D. , Yao, H. , Yoshikawa, H. , Cerezo, A. , Leckman, J. F. , & Bhutta, Z. A. (2017). Nurturing care: Promoting early childhood development. The Lance, 389(10064), 91–102. 10.1016/S0140-6736(16)31390-3 27717615

[cch13138-bib-0017] Brown, T. W. , van Urk, F. C. , Waller, R. , & Mayo‐Wilson, E. (2014). Centre‐based day care for children younger than five years of age in low‐ and middle‐income countries. Cochrane developmental, psychosocial and learning problems group, editor. Cochrane Database of Systematic Reviews, (9), CD010543. https://doi.wiley.com/10.1002/14651858.CD010543.pub2 25254354 10.1002/14651858.CD010543.pub2 PMC10617672

[cch13138-bib-0018] Buss, A. H. , & Plomin, R. (1984). Temperament. Early developing personality traits (Vol. 1). Psychology Press.

[cch13138-bib-0019] Clark, S. , De Almada, M. , Kabiru, C. W. , Muthuri, S. , & Wanjohi, M. (2021). Balancing paid work and child care in a slum of Nairobi, Kenya: The case for centre‐based child care. Journal of Family Studies, 27(1), 93–111. 10.1080/13229400.2018.1511451

[cch13138-bib-0020] Clark, S. , Kabiru, C. W. , Laszlo, S. , & Muthuri, S. (2019). The impact of childcare on poor urban women's economic empowerment in Africa. Demography, 56(4), 1247–1272. 10.1007/s13524-019-00793-3 31286428

[cch13138-bib-0021] Cluver, L. , Rudgard, W. E. , Toska, E. , Orkin, M. , Ibrahim, M. , Langwenya, N. , Kuo, C. , Xaba, N. , Roehm, K. , Smith, M. , Bernardini, S. , Giordana, G. , Mumma, M. , Kingori, J. , Yates, R. , & Sherr, L. (2022). Food security reduces multiple HIV infection risks for high‐vulnerability adolescent mothers and non‐mothers in South Africa: A cross‐sectional study. Journal of the International AIDS Society, 25(8), e25928. 10.1002/jia2.25928 36008916 PMC9411725

[cch13138-bib-0022] Cluver, L. D. , Gardner, F. , & Operario, D. (2007). Psychological distress among AIDS‐orphaned children in urban South Africa. Journal of Child Psychology and Psychiatry, 48(8), 755–763. 10.1111/j.1469-7610.2007.01757.x 17683447

[cch13138-bib-0023] Craig, L. , & Churchill, B. (2018). Parenting stress and the use of formal and informal child care: Associations for fathers and mothers. Journal of Family Issues, 39(12), 3203–3224. 10.1177/0192513X18776419

[cch13138-bib-0024] Crnic, K. A. , Gaze, C. , & Hoffman, C. (2005). Cumulative parenting stress across the preschool period: Relations to maternal parenting and child behaviour at age 5. Infant and Child Development, 14(2), 117–132. 10.1002/icd.384

[cch13138-bib-0025] Deater‐Deckard, K. (1998). Parenting stress and child adjustment: Some old hypotheses and new questions. Clinical Psychology: Science and Practice, 5, 314–332. 10.1111/j.1468-2850.1998.tb00152.x

[cch13138-bib-0026] Devercelli, A. E. , & Beaton‐Day, F. (2020 [cited 2022 Jun 14]). Better jobs and brighter futures: Investing in childcare to build human capital [Internet]. World Bank. Available from: http://elibrary.worldbank.org/doi/book/10.1596/35062

[cch13138-bib-0027] DiCenso, A. , Guyatt, G. , Willan, A. , & Griffith, L. (2002). Interventions to reduce unintended pregnancies among adolescents: Systematic review of randomised controlled trials. BMJ, 324(7351), 1426. 10.1136/bmj.324.7351.1426 12065267 PMC115855

[cch13138-bib-0028] Domond, P. , Orri, M. , Algan, Y. , Findlay, L. , Kohen, D. , Vitaro, F. , Tremblay, R. E. , & Côté, S. M. (2020). Child care attendance and educational and economic outcomes in adulthood. Pediatrics, 146(1), e20193880. 10.1542/peds.2019-3880 32527751

[cch13138-bib-0029] Durkin, M. S. , Wang, W. , Shrout, P. E. , Zaman, S. S. , Hasan, Z. M. , Desai, P. , & Davidson, L. L. (1995). Evaluating a ten questions screen for childhood disability: Reliability and internal structure in different cultures. Journal of Clinical Epidemiology, 48(5), 657–666. 10.1016/0895-4356(94)00163-K 7537327

[cch13138-bib-0030] Engle, P. L. , Fernald, L. C. , Alderman, H. , Behrman, J. , O'Gara, C. , Yousafzai, A. , de Mello, M. C. , Hidrobo, M. , Ulkuer, N. , Ertem, I. , Iltus, S. , & Global Child Development Steering Group . (2011). Strategies for reducing inequalities and improving developmental outcomes for young children in low‐income and middle‐income countries. The Lancet, 378(9799), 1339–1353. 10.1016/S0140-6736(11)60889-1 21944378

[cch13138-bib-0031] Evans, D. K. , Jakiela, P. , & Knauer, H. A. (2021). The impact of early childhood interventions on mothers. Science, 372(6544), 794–796. 10.1126/science.abg0132 34016770

[cch13138-bib-0032] Fall, C. H. D. , Sachdev, H. S. , Osmond, C. , Restrepo‐Mendez, M. C. , Victora, C. , Martorell, R. , Stein, A. D. , Sinha, S. , Tandon, N. , Adair, L. , Bas, I. , Norris, S. , Richter, L. M. , & COHORTS investigators . (2015). Association between maternal age at childbirth and child and adult outcomes in the offspring: A prospective study in five low‐income and middle‐income countries (COHORTS collaboration). The Lancet Global Health, 3(7), e366–e377. 10.1016/S2214-109X(15)00038-8 25999096 PMC4547329

[cch13138-bib-0033] Faul, F. , Erdfelder, E. , Buchner, A. , & Lang, A. G. (2009). Statistical power analyses using G*Power 3.1: Tests for correlation and regression analyses. Behavior Research Methods, 41(4), 1149–1160. 10.3758/BRM.41.4.1149 19897823

[cch13138-bib-0034] Gadoth, A. , & Heymann, J. (2020). Gender parity at scale: Examining correlations of country‐level female participation in education and work with measures of men's and women's survival. EClinicalMedicine., 20, 100299. 10.1016/j.eclinm.2020.100299 32300745 PMC7152805

[cch13138-bib-0035] Gerard, A. B. , & Reynolds, C. R. (1999). Characteristics and applications of the revised children's manifest anxiety scale (RCMAS), The use of psychological testing for treatment planning and outcome assessment (pp. 232–340). Lawrence Erlbaum Associates Publishers.

[cch13138-bib-0036] Greenland, S. , Mansournia, M. A. , & Altman, D. G. (2016). Sparse data bias: A problem hiding in plain sight. BMJ, 352, i1981. 10.1136/bmj.i1981 27121591

[cch13138-bib-0037] Gromada A , Richardson D , Rees G . (2020) Childcare in a global crisis: The impact of COVID‐19 on work and family life [Internet]. [cited 2022 Jun 16]. (Innocenti Research Briefs; vol. 2020/18). Report No.: 2020/18. Available from: https://www.un-ilibrary.org/content/papers/26642166/79

[cch13138-bib-0038] Hall, K. S. , Manu, A. , Morhe, E. , Dalton, V. K. , Challa, S. , Loll, D. , Dozier, J. L. , Zochowski, M. K. , Boakye, A. , & Harris, L. H. (2018). Bad girl and unmet family planning need among Sub‐Saharan African adolescents: The role of sexual and reproductive health stigma. Qualitative Research in Medicine and Healthcare, 2(1), 55–64. 10.4081/qrmh.2018.7062 30556052 PMC6292434

[cch13138-bib-0039] Hallman K , Quisumbing A , Ruel M , de la Briere B . Childcare, mothers' work, and earnings: Findings from the urban slums of Guatemala City [Arabic] [Internet]. Population Council; 2002 [cited 2022 Jun 14]. Available from: https://knowledgecommons.popcouncil.org/departments_sbsr-pgy/306

[cch13138-bib-0040] Horwood, C. , Haskins, L. , Alfers, L. , Masango‐Muzindutsi, Z. , Dobson, R. , & Rollins, N. (2019). A descriptive study to explore working conditions and childcare practices among informal women workers in KwaZulu‐Natal, South Africa: Identifying opportunities to support childcare for mothers in informal work. BMC Pediatrics, 19(1), 382. 10.1186/s12887-019-1737-7 31651267 PMC6814020

[cch13138-bib-0041] Huda, M. M. , O'Flaherty, M. , Finlay, J. E. , & Mamun, A. A. (2021). Time trends and sociodemographic inequalities in the prevalence of adolescent motherhood in 74 low‐income and middle‐income countries: A population‐based study. The Lancet Child & Adolescent Health, 5(1), 26–36. 10.1016/S2352-4642(20)30311-4 33245863

[cch13138-bib-0042] Hughes, R. C. , Kitsao‐Wekulo, P. , Muendo, R. , Bhopal, S. S. , Kimani‐Murage, E. , Hill, Z. , & Kirkwood, B. R. (2021). Who actually cares for children in slums? Why we need to think, and do, more about paid childcare in urbanizing sub‐Saharan Africa. Philosophical Transactions of the Royal Society B, 376(1827), 20200430. 10.1098/rstb.2020.0430 PMC8090813 33938281

[cch13138-bib-0043] Jackson, D. L. (2003). Revisiting sample size and number of parameter estimates: Some support for the N:Q hypothesis. Structural Equation Modeling, 10(1), 128–141.

[cch13138-bib-0044] Jochim J , Cluver LD , Sidloyi L , Kelly J , Ornellas A , Mangqalaza H , et al. (Forthcoming) Improving educational and reproductive outcomes for adolescent mothers in South Africa: A cross‐sectional analysis towards realising policy goals. 10.1080/17441692.2023.2206465 37158293

[cch13138-bib-0045] Kelly J , Ornellas A , Coakley C , Jochim J , Mangqalaza H , Cluver LD , et al. Investing in our future: supporting pregnant and mother learners' return to school|Centre for Social Science Research [Internet]. [cited 2023 Mar 27]. Available from: http://www.cssr.uct.ac.za/cssr/pub/wp/471

[cch13138-bib-0046] Knerr, W. , Gardner, F. , & Cluver, L. (2013). Improving positive parenting skills and reducing harsh and abusive parenting in low‐ and middle‐income countries: A systematic review. Prevention Science, 14(4), 352–363. 10.1007/s11121-012-0314-1 23315023

[cch13138-bib-0047] Kovacs, M. (1985). The Children's Depression Inventory (CDI). Psychopharmacology Bulletin, 21(4), 995–998. 4089116

[cch13138-bib-0048] Kurth, F. , Bélard, S. , Mombo‐Ngoma, G. , Schuster, K. , Adegnika, A. A. , Bouyou‐Akotet, M. K. , Kremsner, P. G. , & Ramharter, M. (2010). Adolescence as risk factor for adverse pregnancy outcome in central Africa—A cross‐sectional study. PLoS ONE, 5(12), e14367. 10.1371/journal.pone.0014367 21188301 PMC3004789

[cch13138-bib-0049] Lester, S. (2015). An evaluation of the parent centre's positive parenting skills training programme: A randomised controlled trial [Internet]. University of Cape Town. Available from: https://open.uct.ac.za/bitstream/handle/11427/15615/thesis_com_2015_lester_soraya_natalie%202.pdf?sequence=1&isAllowed=y

[cch13138-bib-0050] Lipman, E. L. , Georgiades, K. , & Boyle, M. H. (2011). Young adult outcomes of children born to teen mothers: Effects of being born during their teen or later years. Journal of the American Academy of Child and Adolescent Psychiatry, 50(3), 232–241. 10.1016/j.jaac.2010.12.007 21334563

[cch13138-bib-0051] Martinez, S. , Naudeau, S. , & Pereira, V. (2012 [cited 2022 Jun 14]). The promise of preschool in Africa: A randomized impact evaluation of early childhood development in rural Mozambique|3ie [Internet]. 3ie Impact Evaluation Report 001. New Delhi: International Initiative for Impact Evaluation (3ie). Available from: https://3ieimpact.org/evidence-hub/publications/impact-evaluations/promise-preschool-africa-randomized-impact-evaluation

[cch13138-bib-0052] Masi, G. , Mucci, M. , Favilla, L. , Brovedani, P. , Millepiedi, S. , & Perugi, G. (2003). Temperament in adolescents with anxiety and depressive disorders and in their families. Child Psychiatry and Human Development, 33(3), 245–259. 10.1023/A:1021408714741 12564625

[cch13138-bib-0053] Mathiesen, K. S. , & Tambs, K. (1999). The EAS temperament questionnaire—Factor structure, age trends, reliability, and stability in a Norwegian sample. Journal of Child Psychology and Psychiatry, 40(3), 431–439. 10.1111/1469-7610.00460 10190344

[cch13138-bib-0054] McEachern, A. D. , Dishion, T. J. , Weaver, C. M. , Shaw, D. S. , Wilson, M. N. , & Gardner, F. (2012). Parenting young children (PARYC): Validation of a self‐report parenting measure. Journal of Child and Family Studies, 21(3), 498–511. 10.1007/s10826-011-9503-y 22876108 PMC3412343

[cch13138-bib-0055] Mchunu, G. , Peltzer, K. , Tutshana, B. , & Seutlwadi, L. (2012). Adolescent pregnancy and associated factors in South African youth. African Health Sciences, 12(4), 426–434. 10.4314/ahs.v12i4.5 23515418 PMC3598281

[cch13138-bib-0056] Mmari, K. , & Sabherwal, S. (2013). A review of risk and protective factors for adolescent sexual and reproductive health in developing countries: An update. The Journal of Adolescent Health, 53(5), 562–572. 10.1016/j.jadohealth.2013.07.018 23998849

[cch13138-bib-0057] Mullen, E. M. (1995). Mullen scales of early learning. AGS Circle Pines.

[cch13138-bib-0058] Mutahi, J. , Larsen, A. , Cuijpers, P. , Peterson, S. S. , Unutzer, J. , McKay, M. , et al. (2022 1 [cited 2022 Jun 14];44). Available from:Mental health problems and service gaps experienced by pregnant adolescents and young women in Sub‐Saharan Africa: A systematic review. eClinicalMedicine, 44, 101289. https://www.thelancet.com/journals/eclinm/article/PIIS2589-5370(22)00019-0/fulltext, 10.1016/j.eclinm.2022.101289 35198916 PMC8851289

[cch13138-bib-0059] Mwaura, P. A. M. , Sylva, K. , & Malmberg, L. (2008). Evaluating the Madrasa preschool programme in East Africa: A quasi‐experimental study. International Journal of Early Years Education, 16(3), 237–255. 10.1080/09669760802357121

[cch13138-bib-0060] Nakahara, S. , Poudel, K. C. , Lopchan, M. , Poudel, O. R. , Poudel‐Tandukar, K. , & Ichikawa, M. (2010). Differential effects of out‐of‐home day care in improving child nutrition and augmenting maternal income among those with and without childcare support: A prospective before–after comparison study in Pokhara, Nepal. Health Policy, 97(1), 16–25. 10.1016/j.healthpol.2010.02.011 20236723

[cch13138-bib-0061] National Institute of Child Health and Human Development Early Child Care Research Network , & Duncan, G. J. (2003). Modeling the impacts of child care quality on children's preschool cognitive development. Child Development, 74(5), 1454–1475. 10.1111/1467-8624.00617 14552408

[cch13138-bib-0062] Oringanje, C. , Meremikwu, M. M. , Eko, H. , Esu, E. , Meremikwu, A. , & Ehiri, J. E. (2016). Interventions for preventing unintended pregnancies among adolescents. Cochrane fertility regulation group, editor. Cochrane Database of Systematic Reviews, 2(2), CD005215. Available from: http://doi.wiley.com/10.1002/14651858.CD005215.pub3 26839116 10.1002/14651858.CD005215.pub3 PMC8730506

[cch13138-bib-0063] Oxford, M. , & Spieker, S. (2006). Preschool language development among children of adolescent mothers. Journal of Applied Developmental Psychology, 27(2), 165–182. 10.1016/j.appdev.2005.12.013 16619087 PMC1440306

[cch13138-bib-0064] Ramos, S. (2011). Interventions for preventing unintended pregnancies among adolescents. World Health Organization.

[cch13138-bib-0065] Rasbash, J. , O'Connor, T. , & Jenkins, J. (2009). Multilevel models for family data. Bristol, England: Centre for Multilevel Modelling, University of Bristol.

[cch13138-bib-0066] Reynolds, C. R. , & Richmond, B. O. (1978). What I think and feel: A revised measure of children's manifest anxiety. Journal of Abnormal Child Psychology, 6(2), 271–280. 10.1007/BF00919131 670592

[cch13138-bib-0067] Richardson, R. A. , Harper, S. , Schmitz, N. , & Nandi, A. (2018). The effect of affordable daycare on women's mental health: Evidence from a cluster randomized trial in rural India. Social Science & Medicine, 1982(217), 32–41. 10.1016/j.socscimed.2018.09.061 PMC9202395 30292875

[cch13138-bib-0068] Richter, L. M. , Daelmans, B. , Lombardi, J. , Heymann, J. , Boo, F. L. , Behrman, J. R. , Lu, C. , Lucas, J. E. , Perez‐Escamilla, R. , Dua, T. , Bhutta, Z. A. , Stenberg, K. , Gertler, P. , & Darmstadt, G. L. (2017). Paper 3 working group and the lancet early childhood development series steering committee investing in the foundation of sustainable development: Pathways to scale up for early childhood development. The Lancet, 389(10064), 103–118. 10.1016/S0140-6736(16)31698-1 PMC5880532 27717610

[cch13138-bib-0069] Samman, E. , Presler‐Marshall, E. , Jones, N. , Bhatkal, T. , Melamed, C. , Stavropoulou, M. , et al. (2016). Women's work. Mothers, children and the global childcare crisis. Overseas Development Institute.

[cch13138-bib-0070] Sanfelice, V. (2019). Universal public childcare and labor force participation of mothers in Brazil. In Essays on puplic policies using neighborhoods variation. University of Rochester.

[cch13138-bib-0071] Schultz, T. P. (2002). Why governments should invest more to educate girls. World Development, 30(2), 207–225. 10.1016/S0305-750X(01)00107-3

[cch13138-bib-0072] Shafiq, M. N. , Devercelli, A. , & Valerio, A. (2018). Are there long‐term benefits from early childhood education in low‐ and middle‐income countries? Education Policy Analysis Archives, 26, 122. 10.14507/epaa.26.3239

[cch13138-bib-0073] Sharain, S. (2002). Assessing post‐traumatic responses among South African adolescents: A comparison of different methods. [Master's thesis]. [Cape Town]:. University of Cape Town.

[cch13138-bib-0074] Sheehan, D. , Lecrubier, Y. , Harnett Sheehan, K. , Janavs, J. , Weiller, E. , Keskiner, A. , Schinka, J. , Knapp, E. , Sheehan, M. F. , & Dunbar, G. C. (1997). The validity of the mini international neuropsychiatric interview (MINI). European Psychiatry, 12(5), 232–241. 10.1016/S0924-9338(97)83297-X

[cch13138-bib-0075] Sheehan, D. V. , Sheehan, K. H. , Shytle, R. D. , Janavs, J. , Bannon, Y. , Rogers, J. E. , Milo, K. M. , Stock, S. L. , & Wilkinson, B. (2010). Reliability and validity of the mini international neuropsychiatric interview for children and adolescents (MINI‐KID). The Journal of Clinical Psychiatry, 71(3), 313–326. 10.4088/JCP.09m05305whi 20331933

[cch13138-bib-0076] Shenderovich, Y. , Boyes, M. E. , Esposti, M. D. , Casale, M. , Toska, E. , Roberts, K. J. , & Cluver, L. (2021). Relationships with caregivers and mental health outcomes among adolescents living with HIV: A prospective cohort study in South Africa. BMC Public Health, 21(1), 1–11, 172. 10.1186/s12889-020-10147-z 33472607 PMC7816135

[cch13138-bib-0077] Sherr, L. , Croome, N. , Clucas, C. , & Brown, E. (2014). Differential effects of single and double parental death on child emotional functioning and daily life in South Africa. Child Welfare, 93(1), 149–172. 26030991

[cch13138-bib-0078] Steventon Roberts, K. , Smith, C. , Toska, E. , Cluver, L. D. , Wittesaele, C. , Langwenya, N. , Shenderovich, Y. , Saal, W. , Jochim, J. , Chen‐Charles, J. , Marlow, M. , & Sherr, L. (2022). Exploring the cognitive development of children born to adolescent mothers in South Africa. Infant and Child Development, e2408. 10.1002/icd.2408 PMC10909423 38439906

[cch13138-bib-0079] Thurman, T. R. , Brown, L. , Richter, L. , Maharaj, P. , & Magnani, R. (2006). Sexual risk behavior among South African adolescents: Is orphan status a factor? AIDS and Behavior, 10(6), 627–635. 10.1007/s10461-006-9104-8 16838071

[cch13138-bib-0080] UN WOMEN . (2015 [cited 2022 Jun 14]). Gender equality, child development and job creation: How to reap the ‘triple dividend’ from early childhood education and care services [Internet]. UN Women. Available from: https://www.unwomen.org/en/digital-library/publications/2015/12/gender-equality-child-development-job-creation

[cch13138-bib-0081] UNFPA . (2022). Motherhood in childhood—The untold story. UNFPA.

[cch13138-bib-0082] Vandemoortele, J. , & Delamonica, E. (2000). The_education_vaccine_against_HIV.pdf. Current Issues in Comparative Education, 3(1), 6–13.

[cch13138-bib-0083] Wolf, S. , Aber, L. , & Behrman, J. (2019 [cited 2022 Sep 5]). The impacts of teacher training and parental engagement on kindergarten quality in Ghana [Internet]. Results brief (Education). Ghana: Innovations for Poverty Action. Available from: https://www.poverty-action.org/sites/default/files/publications/QP4G-Final-Results-Brief-Updated.pdf

[cch13138-bib-0084] Woollett, N. , Cluver, L. D. , Bandeira, M. , & Brahmbhatt, H. (2017). Identifying risks for mental health problems in HIV positive adolescents accessing HIV treatment in Johannesburg. Journal of Child and Adolescent Mental Health, 29(1), 11–26. 10.2989/17280583.2017.1283320 28287023

[cch13138-bib-0085] World Health Organization . (2020 [cited 2022 Aug 10]). Adolescent pregnancy: WHO factsheet [Internet]. World Health Organization. Available from: https://www.who.int/news-room/fact-sheets/detail/adolescent-pregnancy

[cch13138-bib-0086] Zulaika, G. , Bulbarelli, M. , Nyothach, E. , van Eijk, A. , Mason, L. , Fwaya, E. , Obor, D. , Kwaro, D. , Wang, D. , Mehta, S. D. , & Phillips‐Howard, P. A. (2022). Impact of COVID‐19 lockdowns on adolescent pregnancy and school dropout among secondary schoolgirls in Kenya. BMJ Global Health, 7(1), e007666. 10.1136/bmjgh-2021-007666 PMC8761596 35027438

